# The effectiveness and side effects of conformal external beam radiotherapy combined with high-dose-rate brachytherapy boost compared to conformal external beam radiotherapy alone in patients with prostate cancer

**DOI:** 10.1186/s13014-015-0366-z

**Published:** 2015-03-07

**Authors:** Beata Smolska-Ciszewska, Leszek Miszczyk, Brygida Białas, Marek Fijałkowski, Grzegorz Plewicki, Marzena Gawkowska-Suwińska, Monika Giglok, Katarzyna Behrendt, Elżbieta Nowicka, Aleksander Zajusz, Rafał Suwiński

**Affiliations:** Maria Skłodowska-Curie Memorial Cancer Center and Institute of Oncology, Gliwice Branch, Ul. Wybrzeże Armii Krajowej 15, Gliwice, 44-100 Poland

**Keywords:** Brachytherapy, High-dose-rate, Prostate cancer, Radiotherapy

## Abstract

**Background:**

Clinical data that compare external-beam radiotherapy (EBRT) combined with high-dose-rate brachytherapy (HDR-BT) boost versus EBRT alone are scarce. The analysis of published studies suggest that biochemical relapse-free survival in combined EBRT and HDR-BT may be superior compared to EBRT alone. We retrospectively examined the effectiveness and tolerance of both schemes in a single center study.

**Methods:**

Between March 2003 and December 2004, 229 patients were treated for localized T1-T2N0M0 prostate cancer. Median age was 66 years (range, 49 – 83 years). PSA level ranged from 0.34 to 64 ng/ml (median 12.3 ng/ml) and Gleason score ranged from 2 to 10. The analysis included 99 patients who underwent EBRT with HDR-BT (group A) and 130 patients who were treated with EBRT alone (group B).

**Results:**

Median follow-up was 6 years. Biochemical relapses occurred in 34% vs. 22% (p = 0.002), local recurrences in 17% vs. 5% (p = 0.002), and distant metastases in 11% vs. 6% (p = 0.179) of patients in groups A and B, respectively. Five-year biochemical relapse-free survival was 67% vs. 81% (p = 0.005), local recurrence-free survival 95% vs. 99% (p = 0.002), metastases-free survival 95% vs. 94% (p = 0.302) for groups A and B, respectively. Five-year overall survival was 85% in both groups (p = 0.596). Grade 2/3 late GI complications appeared in 9.2% and 24.8% (p = 0.003), respectively. Grade 2/3 late GU symptoms occurred in 12% in both groups.

**Conclusions:**

Although because of the retrospective character of the study and nonrandomized selection of fractionation schedule the present conclusions had limitations EBRT alone appeared more effective than EBRT combined with HDR-BT. It was likely the result of the less frequent use of androgen deprivation therapy (ADT) for combined scheme group, too low dose in a single BT fraction or inadequate assumptions regarding fractionation sensitivity of prostate cancer.

## Background

Radical prostatectomy (RP) and radiotherapy are two basic methods of curative treatment for prostate cancer (PC). Radiation treatment can be conducted using external beam radiotherapy (EBRT) alone, interstitial brachytherapy (BT) alone or may combine both of these methods. Comparison of different treatment methods for early stage prostate cancer: low-dose-rate BT (LDR-BT), EBRT <72 Gy, EBRT >72 Gy, combined LDR-BT and RP indicated that biochemical relapse-free survival (bRFS) was inferior in low-dose EBRT, compared to the other groups in all prognostic subsets and was not improved by use of ADT [1]. A systematic review of treatment methods for PC by Grimm et al. revealed that the decision on treatment method may depend on risk group. In low-risk disease BT alone might be the choice, for intermediate-risk disease the combination of EBRT and BT appears equivalent to BT alone, for high-risk patients combined therapies that incorporate EBRT and BT plus or minus androgen deprivation therapy (ADT) appear favorable [2].

Most of the published reports suggest that α/β ratio for PC is low, PC may be sensitive, thus, to high fractional doses [3,4]. Hypofractionation enables the application of higher single fractions in a shorter overall treatment time. This method is investigational and may be used for EBRT, fractionated stereotactic body radiotherapy and BT [5-8].

Brachytherapy is one of hypofractionated radiotherapy methods. It can not only shorten the overall treatment time and limit the geographical miss, but also enables escalation of the total dose in the target and reduce the dose in surrounding normal tissues. Permanent or temporary implants are used alone in low-risk (LR) PC patients or are combined with EBRT in intermediate (IR) or high-risk (HR) groups [9]. Clinical data that compare toxicity and efficacy of EBRT combined with HDR-BT boost and EBRT alone are scarce [10-16].

A systematic review of radiotherapy for PC by Pieters et al. revealed that the combination of EBRT and HDR-BT resulted in a better bRFS and OS compared to high-dose EBRT or EBRT combined with LDR-BT [17].

In this report we retrospectively evaluate the effectiveness and normal tissue reactions of EBRT combined with HDR-BT boost in comparison to EBRT alone for PC. While due to nonrandomized selection of fractionation schedules such study has apparent limitations it may provide an interesting insight of the clinical outcomes that can be confronted with the outcomes of other published series.

## Methods

### Patients’ characteristics

Between March 2003 and December 2004, 229 patients with T1-T2N0M0 PC were treated. The analysis included 99 patients who underwent EBRT with HDR-BT (group A) and 130 patients treated with EBRT alone to the median dose of 74 Gy (group B). The median age of the group was 66 years (range, 49 – 83 years). Patients treated with EBRT alone were slightly older than those treated with the combined scheme (67 vs. 65, p = 0.046 Mann–Whitney U test). All patients had biopsy-proven adenocarcinoma of the prostate, Gleason score ranged from 2 to 10. Pretreatment prostate-specific antigen (PSA) was known in all cases and ranged from 1.7 to 64 ng/ml (median 12.3 ng/ml). Prior to initiation of treatment, all patients underwent a complete clinical examination and laboratory tests (blood count, liver and renal function). Staging procedures included digital rectal examination (DRE), transrectal ultrasound (TRUS) of the prostate or pelvic magnetic resonance imaging. Distant metastases were excluded by abdomen and pelvis computed tomography (CT) or ultrasound examination, chest X-ray and bone scan. The clinical stage was defined according to 2009 TNM classification system [18]. Patients were divided into risk groups according to D’Amico [19]. The clinical characteristics of the groups are summarized in Table 1. In general, the groups were comparable with respect to major prognostic factors, except for use of adjuvant ADT (further explained below).

**Table 1:** **Characteristics of 229 patients treated with EBRT-BT and EBRT alone for prostate cancer**

**Characteristics**	**Total**	**Group A** ***EBRT-BT***	**Group B** ***EBRT***	**p (Mann–Whitney U test)**
	**(N =229)**	**(N =99)**	**(N =130)**	
***Age [years]***				0.046
**Median**	66	65	67	
**Range**	49-83	49-83	51-80	
***Zubrod score***				0.1
**0**	196 (85.6%)	89 (89.9%)	107 (82.3%)	
**1**	32 (14%)	10 (10.1%)	22 (16.9%)	
**2**	1 (0.4%)	0 (0%)	1 (0.8%)	
***PSA [ng/ml]***				0.463
**Mean**	16.3	15.4	16.9	
**Median**	12.3	11.8	13.9	
**Range**	1.7-64	3.9-56.6	1.7-64	
***PSA[ng/ml]***				0.962
**<10**	81 (35.4%)	33 (33.3%)	48 (36.9%)	
**10-20**	91 (39.7%)	44 (44.4%)	47 (36.2%)	
**>20**	57 (24.9%)	22 (22.2%)	35 (26.9%)	
***Gleason score***				0.122
**2-6**	115 (67.7%)	67 (67.7%)	88 (67.7%)	
**7**	39 (17%)	18 (18.2%)	21 (16.2%)	
**8-10**	22 (9.6%)	8 (8.1%)	14 (10.8%)	
**unknown**	13 (5.6%)	6 (6%)	7 (5.3%)	
***Clinical T stage***				0.118
**T1b**	8 (3.5%)	2 (2%)	6 (4.6%)	
**T1c**	95 (41.7%)	39 (39.4%)	56 (43.1%)	
**T2a**	49 (21.3%)	19 (19.2%)	30 (23.1%)	
**T2b**	26 (11.3%)	12 (12.1%)	14 (10.7%)	
**T2c**	51 (22.2%)	27 (27.3%)	24 (18.5%)	
***Risk group***				0.310
**LR**	48 (21%)	18 (18.2%)	30 (23.1%)	
**IR**	82 (35.8%)	35 (35.3%)	47 (36.1%)	
**HR**	99 (43.2%)	46 (46.5%)	53 (40.8%)	
***ADT***				
**Neoadjuvant**	206 (90%)	88 (89.8%)	118 (90.7%)	0.639
**Adjuvant**	97 (42.4%)	32 (32.3%)	65 (50%)	0.008
**Salvage**	63 (27.4%)	37 (37.4%)	26 (20%)	0.004
**no**	11 (4.8%)	4 (4%)	7 (5.3%)	
***Total duration of ADT [months]***				<0.001
**Median**	98.9	6	13	
**Range**	0.5-79	0.7-55.6	0.5-79	
***Whole pelvis EBRT***	84 (36.7%)	40 (40.4%)	44 (33.9%)	0.309
***PSA nadir [ng/ml]***				0.049
**Median**	0.04	0.07	0.03	
**Range**	0-12.3	0-12.3	0-3.1	
***Time to obtain PSA nadir [months]***				0.007
**Median**	6.3	5.3	8.7	
**Range**	0.9-74.9	1.1-58.4	0.9-74.9	

The combined treatment was considered in men with good performance status and with prostate suitable for TRUS-guided implantation. Patients with a history of transurethral resection or with PC in clinical T3 stage according to the brachytherapy protocol were excluded. The decision on treatment method – EBRT alone or combined scheme was made by patients. They were informed about treatment methods, all possible complications, the advantages and disadvantages of the proposed schedules and were given liberty to choose a preferred therapy.

### Androgen deprivation therapy

Neoadjuvant ADT was used in 90% patients in both groups. Adjuvant ADT was applied in 97 men: 32/99 (32%) in group A and 65/130 (50%) in group B (p = 0.008 Mann–Whitney U test). Median duration of ADT in group A was 5.9 months compared to 12.9 months in group B (p < 0.001 Mann–Whitney U test). The characteristics of ADT is summarized in Table 1.

### External beam radiotherapy

All patients underwent EBRT with 6–20 MV photons generated by a linear accelerator. Three-dimensional conformal planning technique was based on CT in all cases. Patients were placed in a supine position. Precise and reproducible patient immobilization was achieved using thermoplastic mask system (Orfit Industries, Belgium). Clinical target volume (CTV) included the prostate and base of seminal vesicles, and pelvic lymph nodes in most of HR patients. Planning target volume (PTV) expansion of 10–15 mm was applied, except for the posterior expansion of 7–8 mm. Organs at risk were rectum, urinary bladder, femoral heads and bowel.

Radiation dose was prescribed and specified in the reference point according to the guidelines of the International Commission on Radiation Units Report 50 and 62. Treatment plans were developed with Eclipse treatment-planning system (Varian Medical System, Palo Alto, CA, USA). Varian Clinac linear accelerators (Varian Medical System, Palo Alto, CA, USA) were used in radiation treatment. Portal vision and in vivo dosimetry were used to control the referral points of the dosimetric parameters. The final decision on whole pelvis irradiation depended on risk factors and oncologist’s decision (40/99 (40.4%) in group A and 44/130 (33.9%) in group B received pelvic RT). Median external beam dose to the pelvis was 44 Gy (range, 44–50 Gy) in 22 fractions (range, 22–25 fractions). Median dose to the prostate was 74 Gy (range, 70–76 Gy) in 37 fractions (range, 35–39 fractions) in group B and 54 Gy (range, 51–56 Gy) in 27 fractions (range, 27–29 fractions) in group A. The total duration of radiation treatment in group A was shorter : 34–58 days (median 41) vs. 41–79 days (median 52) in groups A and B, respectively.

### Brachytherapy

Patients were placed in dorsal lithotomic position under spinal or general anesthesia. A Foley catheter was used to fill the urinary bladder with 150 ml of sterile water. A biplaner TRUS was performed to view the prostate, seminal vesicles, urethra, bladder and rectum and prepare a preplan. TRUS probe was fixed to the stepper. The entire prostate gland was defined as clinical target volume (CTV) for HDR-BT. Treatment planning using PLATO Complete (Nucletron, Veenendaal, The Netherlands) was performed with the following constraints: maximum dose to urethra and rectum had to be less than 120% and 7 Gy, respectively. Needles (from nine to eighteen) were implanted under the TRUS guidance. The location of each needle was visualized and the implant was reconstructed by the treatment planning system. A single fraction of 10 Gy was applied by a HDR 192-Ir stepping source unit (MicroSelectron™, Nucletron, Veenendaal, The Netherlands). The dose was calculated on the prostate capsule. A single-fraction HDR-BT boost was given before, during or after EBRT. The date of the procedure depended on patient performance status or waiting time in Brachytherapy Department.

### Follow-up and statistical analysis

Median follow-up time was 6 years (range 0.5–7.6). It was 5.9 years (range, 0.6 – 7.7 years) and 6 years (range, 1–7.6 years) for EBRT alone and EBRT-BT, respectively. Patients were evaluated every 3 months during the first year and every 6 months thereafter. Each evaluation included a clinical examination, DRE and PSA concentration. Acute and late radiation toxicity was evaluated according to the European Organization for Research and Treatment of Cancer / Radiation Therapy Oncology Group (EORTC/RTOG) scoring system [20].

Biochemical failure after RT was defined according to the Radiation Therapy Oncology Group (RTOG) – American Society of Therapeutic Radiology and Oncology (ASTRO) Phoenix consensus criteria: PSA nadir + 2 ng/ml [21]. Local recurrence was defined as local progression detected by a biopsy. The biopsy was performed in patients with biochemical relapse and no evidence of distant metastases. Metastases-free survival (MFS) reflected all distant failures. Overall survival (OS) reflected all deaths, both cancer related or not. RFS and bRFS were calculated from the end of the treatment to the time when any recurrence was detected or to the last follow-up visit. MFS and OS were calculated from the beginning of the treatment to a distant failure, death or the last follow-up visit. The actuarial rates of bRFS, RFS, MFS and OS were estimated according to the Kaplan-Meier method. The statistical significance of the differences between actuarial curves was calculated using the log-rank test. The Cox proportional hazard regression analysis was applied in the multivariable models. Student t or Mann–Whitney U tests were applied to test of the hypothesis that two populations are the same, against an alternative hypothesis. The difference was considered significant if the p value was less than 0.05.

## Results

### PSA nadir

The median nadir value of PSA was lower in group B compared to group A (0.03 vs. 0.07 ng/ml, p = 0.049 Mann–Whitney U test). The time to obtain PSA nadir in the group B was significantly longer (8.7 vs. 5.2 months, p = 0.007 Mann–Whitney U test).

### Treatment outcome

At the median follow-up of 6 years, 56/229 (24%) biochemical relapses, 23/229 (10%) local recurrences and 19/229 (8%) distant metastases occurred in both groups. Biochemical recurrences were significantly more frequent in group A (34/99 (34%) vs. 22/130 (17%) in group B, p = 0.002 Mann–Whitney U test). Local recurrences also occurred more often in group A (17/99 (17%) vs. 6/130 (5%), p = 0.002 Mann–Whitney U test). Distant metastases appeared in 11/99 (11%) patients in group A and in 8/130 (6%) in group B (p = 0.179, Mann–Whitney U test). The most common sites of metastases were bones (73%), lymph nodes (31%), lungs (16%) and the liver (5%).

To date, death occurred in 44/229 (19%) patients in both groups: 17/99 (17%) died in group A and 27/130 (21%) in group B (p = 0.495, Mann–Whitney U test). Ten patients (4%) died of prostate cancer: 5/99 (5%) in group A and 5/130 (4%) in group B (p = 0.659, Mann–Whitney U test). The remaining 34 patients (15%) died due to other causes.

**Figure 1:**
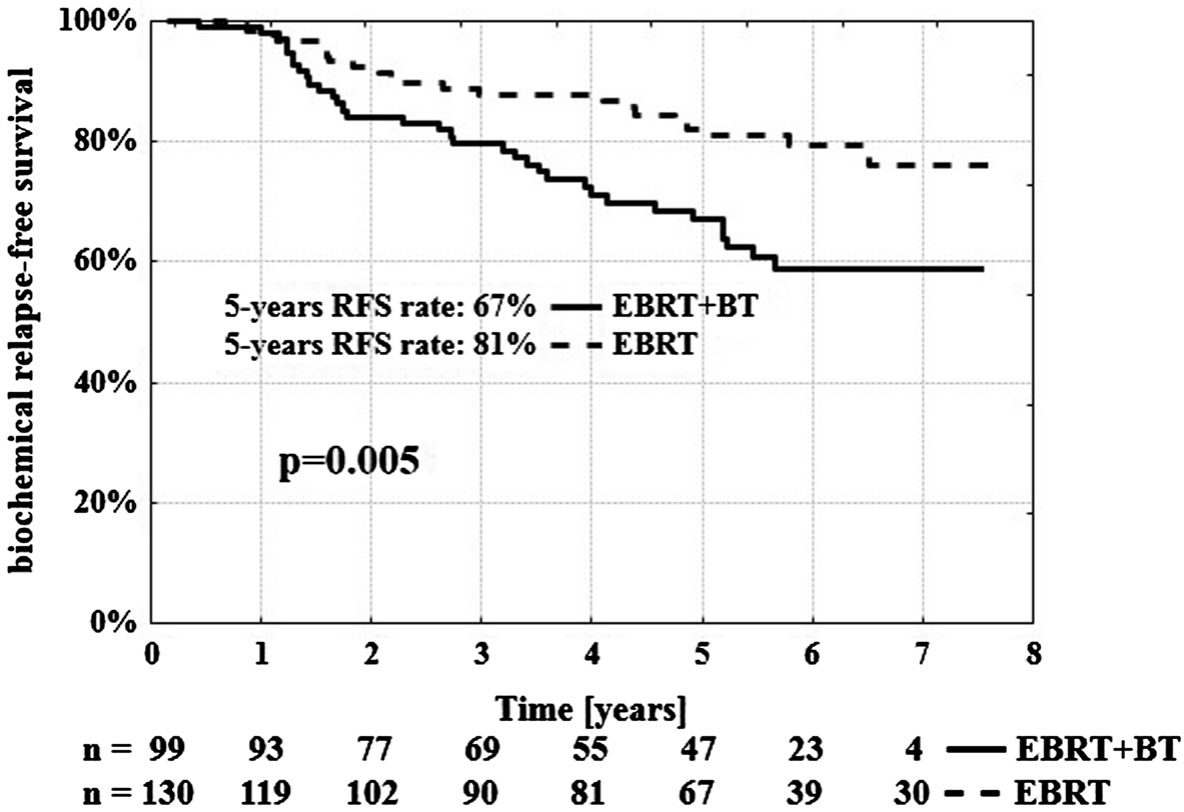
**Biochemical relapse-free survival rate according to treatment schedule.** n-number of patients at risk.

The 5-year bRFS rate for group B was 81% and 67% for group A (p = 0.005, log-rank test) (Figure 1). In the univariable analysis treatment scheme (p = 0.005) and risk group (p = 0.041) were significant predictors of the risk of biochemical failure (Table 2). In the multivariable Cox proportional hazard regression analysis the treatment scheme (p = 0.016) appeared as an independent predictor of the risk of biochemical relapse.

**Table 2:** **Variables influencing actuarial rates of bRFS, RFS, MFS, OS**

**Characteristic**	**5-y bRFS**	**p**	**5-y RFS**	**p**	**5-y MFS**	**p**	**5-y OS**	**p**
***Treatment scheme***		0.005		0.002		0.302		0.596
**EBRT-BT**	67		95		95		85	
**EBRT**	81		99		94		85	
***Age-median***		0.452		0.421		0.948		0.029
**<66**	70		95		95		90	
**≥66**	80		98		94		82	
***Risk group***		0.041		0.894		0.003		0.369
**LR**	83		97		98		85	
**IR**	81		98		100		88	
**HR**	65		96		88		84	
***Total duration of ADT [months]***		0.089		0.748		0.555		0.156
**<9.2**	72		100		93		83	
**≥9.2**	78		96		95		86	

**Figure 2:**
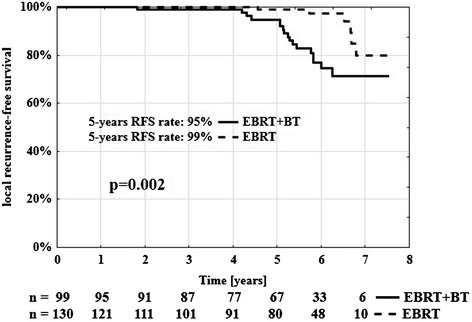
**Local recurrence-free survival rate according to treatment schedule.** n-number of patients at risk.

The 5-year RFS rate was 99% for group B and 95% for group A (p = 0.002, log-rank test) (Figure 2). In the univariable analysis treatment schedule (p = 0.002) was found to be associated with local failure (Table 2). The multivariable Cox proportional hazard regression analysis also showed that treatment schedule (p = 0.045) was associated with higher RFS.

Five-year MFS was 95% and 94% for groups A and B, respectively (p = 0.302, log-rank test). Five-year OS was 85% in both groups (p = 0.596, log-rank test). The Cox proportional hazard regression analysis revealed that risk group (p = 0.049) was significant for prediction of MFS and patient’s age (p = 0.038) was significant for prediction of OS. We also estimated the survival within each risk group. The results are summarized in Table 3.

**Table 3:** **Five-year survivals according to risk groups and treatment methods**

		**LR group**	**p**	**IR group**	**p**	**HR group**	**p**
		**(N = 48)**		**(N = 82)**		**(N = 99)**	
**5-y bRFS**	**EBRT-BT**	77%	0.16	73%	0.06	59%	0.1
	**EBRT**	88%		88%		71%	
**5-y RFS**	**EBRT-BT**	94%	0.07	96%	0.03	94%	0.13
	**EBRT**	100%		100%		100%	
**5-y MFS**	**EBRT-BT**	100%	0.42	100%	0.15	88%	0.98
	**EBRT**	96%		100%		87%	
**5-y OS**	**EBRT-BT**	89%	0.31	88%	0.66	82%	0.72
	**EBRT**	83%		87%		85%	

### Side effects

Grade 2 and 3 acute gastrointestinal (GI) symptoms occurred in 18/99 (18.2%) in group A and in 36/130 (27.7%) in group B (p = 0.094, Mann–Whitney U test). Grade 2 and 3 acute genitourinary (GU) reactions were observed in 42/99 (42.4%) and in 43/130 (33.1%) (p = 0.148, Mann–Whitney U test), respectively. None of the patients developed grade 4 GI or GU early complications. The most frequent acute reactions during radiotherapy were diarrhea of low or moderate severity and polyuria. Six patients required treatment interruption because of severe diarrhea and paralytic subileus.

Grade 2 late GI side effects appeared in 9/98 (9.2%) cases in group A and grade 2 and 3 late GI complications in 31/125 (24.8%) patients in group B (p = 0.003, Mann–Whitney U test). No patient developed grade 4 GI late symptoms. The most frequent late reactions were chronic diarrhea and intermittent rectal bleedings. Four patients required the treatment of argon plasma laser coagulation in group B. Grade 2 and 3 late GU symptoms occurred in 12% in both groups (12/98 in group A and 15/125 in group B). Urethral strictures and hemorrhagic cystitis caused a need for catheterization, required endourethral incisions, transurethral resection of the prostate or electrocoagulation of teleangiectasis, which appeared in 3/98 (3.1%) in group A and in 5/125 (4%) in group B. No patient developed grade 4 GU late complications. The median time interval for occurrence of late GI and GU reactions was 22 months. Both GI and GU late side effects occurred earlier in patients treated with EBRT alone.

## Discussion

This study compares two radiotherapy schedules: EBRT combined with HDR-BT and EBRT alone for patients with PC. After median follow-up time of 6 years, significant differences in clinical outcomes were found between the groups.

The literature data provided by Hoskin et al., Guix et al., Zwahlen et al., Kestin et al. [10-14,16] (Table 4) demonstrated better bRFS after EBRT plus HDR-BT, compared to EBRT alone. Combination of both modalities may also improve OS [17]. Such outcome can be explained by high radiation dose that can be prescribed when HDR-BT is combined with EBRT.

**Table 4:** **Selected published trials on EBRT with HDR-BT in prostate cancer**

**Study**	**scheme**	**dose**	**EQD**_**2**_ **α/β** **= 1.5 Gy**	**EQD**_**2**_ **α/β** **= 3 Gy**	**5-year bRFS**		**5-year OS**
**Hoskin 2007/2012** [10,11] (7-year survival)	**EBRT -BT**	35.75Gy/13fx + 2x8.5Gy	92 Gy	80.21 Gy	75% (66%)		88% (81%)
	**EBRT**	55Gy/20fx	66.8 Gy	63.25 Gy	61% (48%)		89% (81%)
**Guix 2010/2011** [12,13]	**EBRT -BT**	46 Gy/23fx + 2x8Gy	89.4 Gy	81.2 Gy	IR	HR	-
					97%	96%	
	**EBRT**	76 Gy/38fx	76 Gy	76 Gy	90%	89%	-
**Zwahlen 2010 **[14] (7-year survival)	**EBRT -BT**	46 Gy/23fx + 3x6Gy or 4x5Gy	82.1-84.6 Gy	78-78.4 Gy	82.5% (80.3%)		91.9% (89.5%)
	**EBRT**	70 Gy/35fx	70 Gy	70 Gy	81.3% (71%)		88.7% (86.2%)
**Kestin 2000** [16]	**EBRT + HDR-BT**	46 Gy/23fx + 2-3x5.5-10.5Gy	79-118 Gy	74.1-102.7 Gy	67%		-
	**EBRT**	66 Gy/33fx	66 Gy	66 Gy	44%		-
**This study **(7-year survival)	**EBRT + HDR-BT**	54Gy/27fx + 1x10Gy	86.8 Gy	80 Gy	67% (59%)		85% (80%)
	**EBRT**	74Gy/37fx	74 Gy	74 Gy	81% (76%)		85% (73%)

EQD_2_ – equivalent total dose in 2-Gy fractions.

The comparison among the studies presented in Table 4 is difficult due to the apparent differences in the applied dose of EBRT and HDR-BT schemes, diverse definitions of risk groups or biochemical failure. However, we noted that the total dose applied in EBRT alone schedule [10,11,14-16] was lower (66–70 Gy) than recommended by the current guidelines. The adequate dose of 76 Gy in EBRT alone group was applied only in the study by Guix et al. [12,13] (Table 4).

While because of the retrospective character of the study and nonrandomized selection of fractionation schedule the present results have apparent limitations, they suggest relatively low effectiveness of the combined scheme. This finding is inconsistent to previous reports demonstrating the superiority of the combined therapy. Unexpectedly, in spite of initial assumptions based on radiobiological premises i.e.: the prostate tumor’s α/β ratio of 1.5 Gy [22], the scheme was not even as effective as EBRT alone. One may postulate that the assumed α/β ratio value for prostate cancer is not appropriate since some studies suggest is higher value [23-26], including the studies in which HDR brachytherapy was used [27].

Notably, Roberts et al. [27] compared bRFS results for PC patients who received combined EBRT and HDR-BT with expected projections that were based on dose/fractionation/response parameter values derived from EBRT alone. Tumor control rates after EBRT and HDR-BT, were lower in many cases then predicted from linear-quadratic (LQ) parameter estimates from EBRT alone. Lower than expected control rates were associated with BT doses higher than 30 Gy, 1–2 high-dose fractions, 9 fractions (BT alone), doses per fraction of 9–15 Gy, and treatment in only 1 week. It could be explained by cold spots in brachytherapy dose distribution or uncertain applicability of the LQ model at high doses per fraction [27]. This study supports our findings, that a combined scheme of one high-dose BT fraction of 10 Gy may provide biochemical and local control lower than expected.

Relatively small total dose in combined BT and EBRT used in the present study may also contribute to the unsatisfactory local effectiveness. In some studies BT was administered in the minimum of 2 fractions that allowed to escalate the dose, limit the toxicity and avoid the risk of nonhomogenous dose distribution (Table 4). On the other hand, escalation of EBRT dose with one BT fraction may appear effective, as shown by Agoston et al. [28] or Boladeras et al. [29].

Another factor contributing to the observed outcome could be the difference in use of hormone treatment in the analyzed groups: adjuvant ADT was more often applied in the EBRT alone than in the group treated with the combined method (50% vs. 32%, p = 0.008). The total time of the applied ADT was also longer in the EBRT alone (12.9 vs. 5.9 months, p < 0.001). In Agoston et al. and Boladeras et al. studies that show a favorable outcome after single BT boost radiation treatment was accompanied by long adjuvant ADT in most of the patients [28,29].

At last, we note that the bRFS rate in the EBRT alone group in the present study compares favorably to bRFS rates in some published studies on a high-dose radiotherapy [30,31].

The majority of studies on radiotherapy in prostate cancer assessed not only the effectiveness of treatment but also its tolerance. Comparing the toxicity in diverse studies is difficult due to different classifications of radiation reactions or by reporting the incidence of severe side effects only. According to the published studies, acute GU reactions were more intense in the combined scheme groups [8,14,16]. By contrast, late GI reactions more often appeared in EBRT alone groups [12,13]. Late GU side effects were comparable in both groups [10-13,15]. A similar outcome has been presented in this analysis. In general, the treatment was well tolerated in both groups.

## Conclusions

Although because of the retrospective character of the study and nonrandomized selection of fractionation schedule the present conclusions had limitations, EBRT alone appeared more effective than EBRT combined with HDR-BT. It was likely the result of the less frequent use of androgen deprivation therapy in combined scheme group, too low dose in a single BT fraction or inadequate assumptions regarding fractionation sensitivity of prostate cancer. Acute and late GI complications were more severe in the group treated with EBRT alone. Acute GU symptoms were more intense in the EBRT with HDR-BT group, late GU reactions were comparable in both groups.

## Abbreviations

ADT: Androgen deprivation therapy
ASTRO: American Society of Therapeutic Radiology and Oncology
bRFS: Biochemical relapse-free survival
BT: Brachytherapy
CT: Computed tomography
CTV: Clinical target volume
DRE: Digital rectal examination
EBRT: External beam radiotherapy
EORTC: European Organization for Research and Treatment of Cancer
EQD_2_: Equivalent total dose in 2-Gy fractions
Fx: Fraction
Gy: Gray
HDR-BT: High-dose-rate brachytherapy boost
HR: High-risk
IR: Intermediate-risk
LDR: Low-dose-rate
LQ: Linear-quadratic
LR: Low-risk
MFS: Metastases-free survival
OS: Overall survival
PC: Prostate cancer
PSA: Prostate specific antigen
PTV: Planning target volume
RFS: Recurrence-free survival
RP: Radical prostatectomy
RTOG: Radiation Therapy Oncology Group
SBRT: Stereotactic body radiotherapy
TRUS: Transrectal ultrasound
